# Total and acute uterine inversion after delivery: a case report

**DOI:** 10.1186/1752-1947-8-347

**Published:** 2014-10-17

**Authors:** Rui Filipe Monteiro Leal, Rita Mano Luz, José Pinto de Almeida, Vitorino Duarte, Isabel Matos

**Affiliations:** 1 Gynecology Department of Setubal Hospital Center, Rua Camilo Castelo Branco, 2910-446 Setúbal, Portugal

**Keywords:** Hypovolemic shock, Obstetric emergency, Prolapse, Uterine bleeding, Uterine inversion

## Abstract

**Introduction:**

Uterine inversion is a rare obstetric emergency that can lead to hypovolemic shock or even maternal death. There are many management strategies, but they are poorly described and dispersed in the medical literature. The purpose of this article is to describe a case of complete acute uterine inversion and a review of the literature.

**Case presentation:**

The authors describe a case of complete uterine inversion after a normal delivery with fundal placenta and without cord traction, in a 33-year-old Caucasian woman. After the diagnosis was made and after several attempts of manual correction of the inversion, the patient was taken immediately to the operating room and a laparotomy was performed. With opposing pressures in the cervical ring through the abdominal cavity and on the uterus fundus through her vagina, the inversion was resolved. An incision on the cervical ring was unnecessary. Due to incomplete detachment of the placenta the bleeding was mild. She recovered without complications and the histological examination of placenta was unremarkable. In this case, the only risk factor for uterine inversion was the fundal implantation of the placenta.

**Conclusions:**

The low incidence of uterine inversion leads to sparse experience in resolving this obstetrical emergency. The best prognosis occurs in situations where the diagnosis and maneuvers for uterine reversal are made at an early stage. The authors concluded that opposing pressures in the cervical ring through the abdominal cavity and on the uterus fundus through the vagina can resolve the inversion without the need of other surgical techniques. It is essential to keep in mind this diagnosis, and be updated about the strategies required to solve this complication.

## Introduction

Uterine inversion is a rare obstetric emergency. The incidence varies considerably and can range from 1 case in 2000 to 1 case in every 50,000 births [1]. This postpartum complication has an academic importance due to its rarity and severity. When not immediately identified, the massive and often underestimated blood loss can lead to hypovolemic shock and maternal death that can reach 15% in some series [2].

The best management options for this condition are not fully known, given the worldwide scarce experience of each obstetrical team managing this type of situation. There are several therapeutic strategies described in the literature, including drugs, manual maneuvers and surgical interventions.

The aim of this article is to describe a case of complete acute uterine inversion after a normal delivery, and provide a literature review of uterine inversion, its definition, etiology, predictive and risk factors, diagnosis and treatment.

## Case presentation

**Figure 1:**
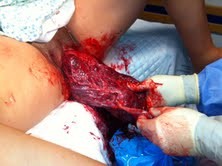
Adherent placenta to the uterine fundus.

**Figure 2:**
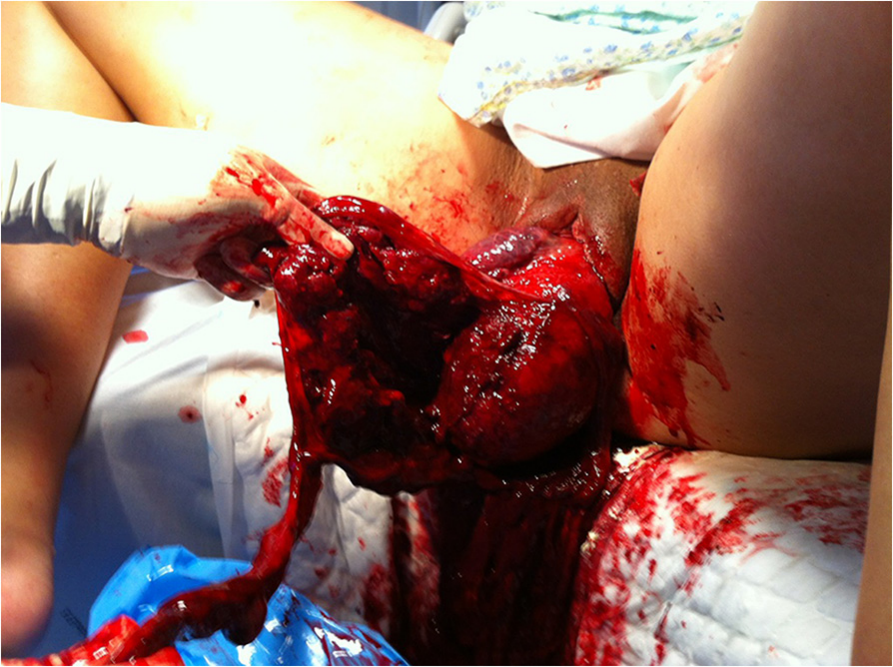
Inverted uterus.

A 33-year-old Caucasian woman 39 weeks’ pregnant was admitted to our hospital in the first stage of labor. She was healthy, nullipara and the pregnancy proceeded without complications. After 6 hours of active phase of labor, she had a normal delivery complicated by shoulder dystocia easily resolved with the McRoberts maneuver. The male neonate weighted 3160g and had an Apgar score of 10 at the 1st minute and 10 at the 5th minute. Twenty minutes after delivery, without any cord traction, the placenta, with fundal implantation, passed through the introitus [Figure 1]. The placenta and membranes were overlaying a firm mass that was identified as the uterine cavity [Figure 2] and the diagnosis of complete acute uterine inversion was made.

Immediately, administration of salbutamol was initiated and the medical team started maneuvers of manual correction of uterine inversion. Due to the unsuccessful attempts, it was decided to try the same technique under general anesthesia, so the patient was taken immediately to the operating room. After another attempt to reposition her uterus, without success, a laparotomy was performed. With opposing pressures in the cervical ring through the abdominal cavity and on the uterus fundus through her vagina, the inversion was resolved. It was not necessary to make an incision in the cervical ring. The incomplete separation of the placenta and membranes contributed to her mild blood loss (estimated at approximately 100cc). Her blood pressure and cardiac frequency were normal during surgery. After the uterine reversion, her uterus and adnexa had normal macroscopic appearance. She recovered from the postoperative period without complications. A histological examination of the placenta had no alterations and no signs of acretism. In this case, there was only one risk factor for uterine inversion: the fundal insertion of the placenta.

## Discussion

### Uterine inversion

#### ***Definition***

Uterine inversion is defined as the passage of the uterine fundus through the endometrial cavity and cervix, turning the uterus inside out. This, although rare, can occur in two distinct clinical situations: in the postpartum period and spontaneously. Non-puerperal uterine inversion accounts for 5% of all uterine inversions and is generally associated with exteriorization of uterine cavity tumors [3,4].

Uterine inversion can be classified in four degrees, depending on the localization of the uterine fundus. In the 1st degree, the fundus is inside the cavity. If it reaches but does not exceed the cervical external os, it is a 2nd degree inversion. A 3rd degree inversion occurs when the fundus extends out of the external os. When it is beyond the vaginal introitus, it is called complete inversion [5] or 4th degree uterine inversion [6].

The term full uterine inversion is used to report situations of vaginal and uterine inversion caused by mass effect in the context of a pelvic tumor; it is also important to describe when the inversion occurs in relation to delivery. Thus it is classified as acute uterine inversion if it takes place before the contraction of the cervical ring, subacute if this occurs after contraction of the cervical ring, or chronic if it occurs after the first 4 weeks after birth [7].

#### ***Etiology***

Although generally associated with excessive cord traction in the third stage of labor, the causes of uterine inversion remain unexplained. In fact, there are several cases in which no tension was carried out on the cord. Risk factors associated with this situation are tension on the umbilical cord, fetal macrosomia, excessive fundal pressure, placenta accreta, short umbilical cord, ligaments laxity, and congenital abnormalities of the uterus [2,8].

Some authors argue that the use of magnesium sulfate may be a risk factor for uterine inversion, although there is no scientific evidence to support this. Some studies have suggested that the rapid uterine emptying, nulliparity and fundal implantation of the placenta are other predisposing factors for uterine inversion.

#### ***Diagnosis***

The diagnosis of uterine inversion is clinical. The observation of the uterine fundus beyond the vaginal introitus in the complete form or the palpation of the fundus through the external os in the 3rd degree uterine inversion is the most common sign. Nevertheless, the diagnosis is often suspected in the presence of massive blood loss after childbirth or in the absence of uterine fundus during abdominal palpation. Hypotension and tachycardia may supervene and evolve into hypovolemic shock. When a physical examination is inconclusive and the patient is hemodynamically stable, the diagnosis can also be confirmed by ultrasound, which detects a vaginal mass with specific characteristics (the echogenicity of the endometrium shows the shape of C letter and the echogenicity of the uterus the shape of H letter) [9].

#### ***Treatment***

The initial approach is to try to reverse immediately the uterus with manual pressure on the fundus through the vagina. This maneuver, called Johnson maneuver, should be carried out as soon as possible to minimize the blood loss and to improve the chances to resolve, since the longer the time between the inversion and the beginning of the maneuver, the lower is the success rate. This is explained by the involution of the cervix which induces a rigid ring that makes the restoration of the normal position of the uterus difficult. It is also essential to establish other therapeutic actions including suspension of oxytocic infusion and administration of drugs with utero-relaxant effect. Magnesium sulfate and salbutamol are the most commonly prescribed drugs due to their availability and frequent administration. Some authors have reported good results with nitroglycerin for relaxation of the cervical ring [10].

When the initial approach fails, it is essential to have an operating room, an obstetrical team and an anesthetist available for a surgical intervention. There are two main surgical techniques described: Huntington and Haultaim. According to the Huntington technique, clamps are placed on the round ligament, near to its insertion in the uterus, and traction is applied while the assistant exerts traction on the contralateral way through the vagina. This is the simplest technique and has a lower risk of complications. In case of failure, the Haultaim technique should be performed. In this technique, an incision is made in the posterior portion of the ring formed by the cervix in order to increase the size of the ring and thus reposition the uterus. Taking into account the low incidence of uterine inversion not reduced by the Johnson maneuver, there are no cohort studies large enough to establish the individual success rate of these two surgical techniques or randomized controlled trials to compare them. Another surgical technique by vaginal route was described by Spinelli. In the latter, the surgeon performs a dissection of the vesicouterine space and makes an incision on the cervix, allowing the uterus to return to its original position [11].

Some authors described a technique using hydrostatic pressure as an alternative when manual reduction is not successful and conditions for surgical intervention are absent [2,10]. In this technique, balloons are placed intravaginally to increase the pressure on the uterine fundus to push the uterus to its initial position. There are other techniques described, but they still require scientific studies to demonstrate their efficacy and safety, including the use of obstetric vacuum extractor (ventouse) to reverse the uterine fundus [12] or surgical resolution by laparoscopy [13].

Regardless of the technique, there is no consensus on the timing of the removal of the placenta. However, many authors argue that this removal should occur only after the normal repositioning of the uterus, to reduce blood loss [14]. After reversal of the clinical condition, it is essential to administrate uterotonic agents (oxytocin or misoprostol) to prevent recurrence. Some authors support the use of large spectrum antibiotics to prevent endometritis or sepsis [15].

## Conclusions

Uterine inversion is an obstetric complication that, due to its gravity, requires a rapid diagnosis and immediate clinical action. Its low incidence leads to scarce experience in solving this kind of situation. Regardless of the treatment, vaginal or surgical approach, the best prognosis occurs in situations when the diagnosis and maneuvers for uterine reversal are made early. The authors concluded that there are no predictive factors known for uterine inversion because of its rarity, only risk factors. Therefore, it is essential to keep in mind this diagnosis in all cases of postpartum hemorrhage, and be updated about the medical therapy and surgical techniques required to solve this type of complication.

## Consent

Written informed consent was obtained from the patient for publication of this case report and any accompanying images. A copy of the written consent is available for review by the Editor-in-Chief of this journal.

## Competing interests

The authors declare that they have no competing interests.

## Authors’ contributions

RL participated in the creation and writing of the manuscript; RML collaborated on bibliographic research; JPA, VD and IM helped the alignment and revision of the manuscript. All authors read and approved the final manuscript.
